# A Cross-Sectional Study of People with Epilepsy and Neurocysticercosis in Tanzania: Clinical Characteristics and Diagnostic Approaches

**DOI:** 10.1371/journal.pntd.0001185

**Published:** 2011-06-07

**Authors:** Joachim Blocher, Erich Schmutzhard, Patricia P. Wilkins, Paige N. Gupton, Matthias Schaffert, Herbert Auer, Thaddaeus Gotwald, William Matuja, Andrea S. Winkler

**Affiliations:** 1 Department of Neurology, Medical University of Innsbruck, Innsbruck, Austria; 2 Haydom Lutheran Hospital, Mbulu, Manyara Region, Tanzania; 3 Department of Neurology, University Medical Centre Göttingen, Göttingen, Germany; 4 Division of Parasitic Diseases, Center for Global Health, Centers for Disease Control and Prevention, Atlanta, Georgia, United States of America; 5 Department of Medical Parasitology, Institute of Specific Prophylaxis and Tropical Medicine, Medical University Vienna, Vienna, Austria; 6 Department of Radiology, Medical University of Innsbruck, Innsbruck, Austria; 7 Department of Neurology, Muhimbili National Hospital, Dar es Salaam, Tanzania; 8 Department of Neurology, Technical University Munich, Munich, Germany; Universidad Nacional Autónoma de México, México

## Abstract

Neurocysticercosis (NCC) is a major cause of epilepsy in regions where pigs are free-ranging and hygiene is poor. Pork production is expected to increase in the next decade in sub-Saharan Africa, hence NCC will likely become more prevalent. In this study, people with epilepsy (PWE, n = 212) were followed up 28.6 months after diagnosis of epilepsy. CT scans were performed, and serum and cerebrospinal fluid (CSF) of selected PWE were analysed. We compared the demographic data, clinical characteristics, and associated risk factors of PWE with and without NCC. PWE with NCC (n = 35) were more likely to be older at first seizure (24.3 vs. 16.3 years, p = 0.097), consumed more pork (97.1% vs. 73.6%, p = 0.001), and were more often a member of the Iraqw tribe (94.3% vs. 67.8%, p = 0.005) than PWE without NCC (n = 177). PWE and NCC who were compliant with anti-epileptic medications had a significantly higher reduction of seizures (98.6% vs. 89.2%, p = 0.046). Other characteristics such as gender, seizure frequency, compliance, past medical history, close contact with pigs, use of latrines and family history of seizures did not differ significantly between the two groups. The number of NCC lesions and active NCC lesions were significantly associated with a positive antibody result. The electroimmunotransfer blot, developed by the Centers for Disease Control and Prevention, was more sensitive than a commercial western blot, especially in PWE and cerebral calcifications. This is the first study to systematically compare the clinical characteristics of PWE due to NCC or other causes and to explore the utility of two different antibody tests for diagnosis of NCC in sub-Saharan Africa.

## Author Summary

Neurocysticercosis, a preventable and treatable disease, is one of the main causes of epilepsy in low income countries. In these countries, the diagnosis of epilepsy is often based on clinical presentation and interviews as neuroimaging is rarely available. It is crucial to distinguish people with epilepsy due to neurocysticercosis from other people with epilepsy by clinical symptoms and/or serology, because the former warrants a specific approach both in terms of diagnosis and treatment. The authors compared the demographic and clinical data of the two groups and found that people with epilepsy due to neurocysticercosis are older, more likely to consume pork, and respond better to anti-epileptic treatment. Additionally, the authors compared two antibody tests for cysticercosis with computed tomography images, which showed a higher sensitivity of the CDC electroimmunotransfer blot compared to a commercial western blot. The number of neurocysticercosis lesions was significantly associated with a positive antibody result in both tests. In summary, this research describes clinical characteristics of people with epilepsy and neurocysticercosis and assesses the usefulness of two immunoblots in those patients. This has implications not only for the diagnosis of neurocysticercosis in low income countries, but also for future epidemiological research.

## Introduction

### Epilepsy in Low Income Countries

Epilepsy is one of the most common neurological disorders worldwide. More than 80% of people with epilepsy (PWE) live in low income countries [1] and more than 75% of them are not treated sufficiently [2]. The prevalence of epilepsy is two to ten times higher and the incidence rate twice that of high income countries [3]. In northern Tanzania, where our study took place, recent results indicate a prevalence of 11.2 per 1000 [4]. One important step towards reducing the burden of epilepsy is to assess its prevalence, causes and risk factors in resource poor countries.

### Cysticercosis

According to the International League Against Epilepsy (ILAE), neurocysticercosis (NCC) is a growing problem in tropical countries and increasingly recognized as a leading cause of epilepsy [5]–[8]. A recent meta-analysis including only studies from Africa revealed a highly significant association between cysticercosis and epilepsy, suggesting that NCC is a major cause of epilepsy in Africa [9]. In endemic countries, NCC is the cause of epilepsy in more than a quarter of PWE [10]. Worldwide, NCC is the most common parasitic disease of the nervous system [1]. Cysticercosis occurs when humans become infected with *Taenia solium* eggs and develop the larval stage. Typical clinical manifestations of NCC are epileptic seizures, which are caused by the cysticerci themselves and by the host's immune response [11].

Pig farming has increased considerably in East and South Africa, especially in rural, low income, smallholder communities [12]. Thus cysticercosis may represent a growing problem, especially in rural areas like our study area, where proper meat inspection does not exist and lack of health education and hygiene is common [11]. The costs of cysticercosis are considerable. Carabin et al. calculated a loss of 34.2 million US-Dollars caused by cysticercosis for the Eastern Cape Province in South Africa with 7 million inhabitants in 2004 [13].

Diagnostic accuracy is the key to identify people with NCC and to treat them according to established guidelines. In most low income countries where NCC is rampant, neuroimaging is not readily available. Diagnosis is established based on medical history, physical examination and, if present, laboratory testing. In our study, we present clinical details of PWE with and without NCC, results of neuroimaging and of two different antibody tests for cysticercosis in an area of northern Tanzania, which only recently has been shown to be highly endemic for cysticercosis [14], [15].

## Methods

### Ethical Considerations

The project was ethically cleared by The National Institute for Medical Research (NIMR), Tanzania. Patients were informed about the risks and benefits of participating in the study, computed tomography (CT) examination and necessary treatments. Written informed consent was obtained from all participants. Patients received free anti-epileptic and, if necessary, anthelmintic treatment according to national guidelines during and after the study. The shipment of samples to the Centers for Disease Control and Prevention, in Atlanta, GA (CDC), was in accordance with institutional review boards and material transfer agreements between Haydom Lutheran Hospital (HLH), CDC, NIMR and the Medical University Innsbruck.

### Study Site

The study was conducted at HLH, which is situated in a remote rural area of northern Tanzania. The total immediate catchment area of the hospital comprises 316,168 people [16]. In 2002, one of the authors (ASW) established a clinic for PWE at HLH, which nowadays cares for more than 400 PWE. The implementation of CT scanning in 2005 further improved diagnostic capacity for people with epileptic seizures/epilepsy. The main tribes in the study area are Iraqw, Datoga and Bantu tribes. The Iraqw and Bantu are agro-pastoralists, while the Datoga traditionally have been nomadic pastoralists, though many are now less nomadic and more agricultural.

### Recruitment of People with Epilepsy

**Figure 1:**
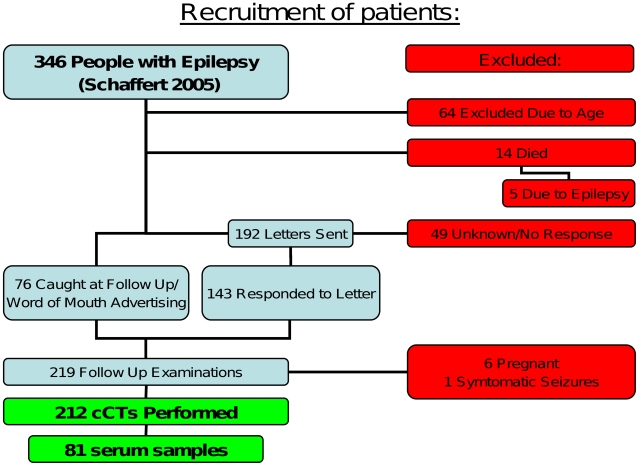
Flow chart for the recruitment of patients. For more details see Methods.

In a former project between August 2002 and October 2004, our research group identified and characterised 346 people with epileptic seizures [17]. Demographic and clinical characteristics, psychosocial as well as sociocultural aspects and the pattern of injuries were evaluated. Epilepsy was defined as two or more afebrile seizures unrelated to acute metabolic disorders or to withdrawal of drugs or alcohol [18] and grouped according to a classification developed for resource poor countries [19], which is based on the ILAE classification for epileptic seizures [20]. The diagnosis was based on seizure semiology, past and present medical history, other associated risk factors and physical examination only. Imaging or EEG was not available at time of diagnosis of epilepsy. Of these 346 people with epileptic seizures 212 participated in the CT study. Due to ethical reasons children younger than ten years at time of diagnosis of epilepsy and pregnant women were excluded. For more details on inclusion and exclusion criteria see Figure 1.

### Study Protocol

All participating PWE were interviewed by a doctor in training and two local nurses using a follow up protocol, which had been validated in a previous study [17]. Additional questions related to NCC were added. Compliance with treatment was assessed by checking regular attendance at HLH and, if attending regularly, calculating the number of tablets that should have remained since the last appointment and counter-checking it with the actual number of tablets.

### Neuroimaging and Serodiagnosis

All 212 PWE underwent CT examination. The CT machine was a Toshiba Auklet Single Slice Spiral CT. The thickness of slices was 5 mm in the posterior cranial fossa and 10 mm for the rest. Intravenous contrast medium was applied in all patients. The pictures were saved digitally and sent to Innsbruck, where a neuroradiologist reviewed all scans. A detailed description of the results on neuroimaging has recently been published [15].

CT based diagnosis of NCC was divided into three groups: definite NCC lesions, lesions highly suggestive of, and those compatible with NCC. Definite NCC lesions were cystic lesions showing the scolex. Any cystic lesion without a visible scolex, single or multiple ring or nodular enhancing lesions and parenchymal brain calcifications were categorized as lesions highly suggestive of NCC [21], [22]. Any pathology that might be caused by NCC such as hydrocephalus or enhancement of the leptomeninges was considered compatible [21], [22], single calcifications in parenchymal brain were also considered compatible. Active NCC was defined as any cystic lesions or lesions with ring enhancement. Parenchymal calcifications were classified as inactive [23], [24].

Due to financial restrictions only part of the collected serum samples (20 of PWE with either highly suggestive or definite NCC lesions and 20 of PWE without NCC lesions on CT scan) and all 11 CSF samples of PWE with highly suggestive or definite NCC lesions on CT scan were analysed with a commercially available western blot (CWB) for cysticercosis (LDBio, Lyon, France) [25]–[27] at the Department for Medical Parasitology, Institute of Specific Prophylaxis and Tropical Medicine, Medical University Vienna, Austria. The antigen used in this test was prepared from *T. solium* larvae (cysticerci). At a later stage of the project all collected serum samples (28 of PWE with either highly suggestive or definite NCC lesions, 7 of PWE with compatible NCC lesions and 46 of PWE without NCC lesions on CT scan) and CSF samples (11 CSF samples of PWE with highly suggestive or definite NCC lesions on CT scan) were tested at the CDC using the CDC-developed enzyme-linked electroimmunotransfer blot (CDC EITB). The CDC EITB was performed as described previously [28].

### Diagnostic Criteria of Neurocysticercosis

The criteria proposed by Del Brutto et al. were used for diagnosis of clinical NCC. A set of defined absolute, major and minor criteria, based on clinical signs and symptoms, neuroimaging, detection of antibodies and epidemiological considerations, were used to establish degrees of certainty for diagnosis, which are classified as definitive and probable NCC [21], [22]. In our study, we compared PWE with definitive or probable NCC to those without NCC. A positive serological result in both tests (CWB and CDC EITB) was only considered once as a criterion for the diagnosis of NCC.

### Statistical Analysis

All data were entered in a SPSS-database. Statistical analysis was performed with the same program. Since all numeric variables had a non-parametric distribution, differences between PWE with and without NCC were tested for significance with Mann-Whitney-U test. Categorical data were tested with Fisher's exact test. McNemar's test was applied to data from samples that were analysed with both antibody tests in order to find significant differences. A p-value lower than 0.05 was regarded as significant. Insufficient answers were left as missing data in the database and were excluded from statistical analysis. Hence the number of people within a specific category might differ from the total number of examined PWE (n = 212).

The reduction of seizure frequency was calculated by dividing the seizure frequency after treatment in 2006 by the seizure frequency before treatment. People with no seizures in 2006 had a reduction of 100 percent. People with an increased number of seizures had zero percent reduction.

## Results

### Demographic Details

The median duration of epilepsy from the day of diagnosis until the day of the follow up for this study was 28.6 months. The range was between 19.3 and 47.7 months. In our cohort of 212 PWE, 35 (16.5%) satisfied the diagnostic criteria for either probable or definitive NCC. The main demographic findings are shown in Table 1.

**Table 1:** Demographic data, clinical characteristics and associated factors of people with epilepsy with and without neurocysticercosis.

Characteristics			PWE with NCC	PWE without NCC	Total
Age at examination day in years		N	35	177	212
p = 0.530		Mean (SD)	32.5 (20.6)	27.3 (12.7)	28.2 (14.4)
		Median (range)	22 (13–83)	24 (11–72)	24 (11–83)
Age at first seizure in years		N	35	176	211
p = 0.097		Mean (SD)	24.3 (22.0)	16.3 (12.2)	17.7 (14.5)
		Median (range)	17 (0.1–77)	14 (0.3–64)	14 (0.1–77)
Gender	Male	N (%)	21 (60.0)	87 (49.2)	108 (50.9)
p = 0.270	Female	N (%)	14 (40.0)	90 (50.8)	104 (49.1)
Tribe	Iraqw	N (%)	33 (94.3)	120 (67.8)	153 (72.2)
**p = 0.005**	Datoga	N (%)	2 (5.7)	27 (15.3)	29 (13.7)
	Bantu tribes	N (%)	0 (0.0)	28 (15.8)	28 (13.2)
	Other	N (%)	0 (0.0)	2 (1.1)	2 (0.9)
Educational level	None	N (%)	14 (40.0)	53 (30.1)	67 (31.8)
p = 0.734	Less than 7 years	N (%)	9 (25.7)	49 (27.8)	58 (27.5)
	Primary school (7 years)	N (%)	12 (34.3)	68 (38.6)	80 (37.9)
	7–11 years	N (%)	0 (0.0)	1 (0.6)	1 (0.5)
	Secondary school (11 years)	N (%)	0 (0.0)	5 (2.8)	5 (2.4)
Type of seizure1)	Gwa	N (%)	15 (42.9)	100 (56.5)	115 (54.2)
p = 0.461	Gfs	N (%)	7 (20.0)	33 (18.6)	40 (18.9)
	Goa	N (%)	8 (22.9)	22 (12.4)	30 (14.2)
	Gbd	N (%)	2 (5.7)	11(6.2)	13 (6.1)
	Tt	N (%)	3 (8.6)	9 (5.1)	12 (5.7)
	U	N (%)	0 (0.0)	2 (1.1)	2 (0.9)
Frequency of seizures / month before Tx		N	26	153	179
p = 0.461		Mean (SD)	6.8 (14.1)	7.8 (19.3)	7.7 (18.6)
		Median (range)	2.75 (0.1–70)	3 (0.1–180)	3 (0.1–180)
Frequency of seizures / month after Tx in 2006		N	35	175	210
p = 0.093		Mean (SD)	0.4 (0.8)	0.8 (2.6)	0.8 (2.4)
		Median (range)	0 (0–3)	0.2 (0–29.5)	0.2 (0–29.5)
Compliance with Tx	Compliant	N (%)	14 (42.4)	95 (53.7)	109 (51.9)
p = 0.259	Non-compliant	N (%)	19 (57.6)	82 (46.3)	101 (48.1)
Reduction of seizure frequency in 2006 in percent		N	32	169	201
All PWE		Mean (SD)	90.2 (21.9)	81.6 (29.5)	82.9 (28.5)
p = 0.081		Median (range)	100 (0–100)	97.8 (0–100)	99.6 (0–100)
Reduction of seizure frequency in 2006 in percent		N	16	80	96
Non compliant		Mean (SD)	82.2 (29.0)	73.0 (34.1)	74.6 (33.3)
p = 0.362		Median (range)	96.5 (0–100)	87.0 (0–100)	89.3 (0–100)
Reduction of seizure frequency in 2006 in percent		N	14	89	103
All Compliant:		Mean (SD)	98.6 (3.6)	89.2 (22.1)	90.5 (20.8)
**p = 0.046**		Median (range)	100 (89–100)	100 (0–100)	100 (0–100)
Reduction of seizure frequency in 2006 in percent		N	9	65	74
Compliant on Carbamazepine		Mean (SD)	99.0 (2.8)	89.2 (21.7)	90.4 (20.6)
p = 0.069		Median (range)	100 (92–100)	98.8 (0–100)	100 (0–100)
Reduction of seizure frequency in 2006 in percent		N	5	21	26
Compliant on Phenobarbitone		Mean (SD)	97.8 (5.0)	87.9 (24.7)	89.8 (22.5)
p = 0.294		Median (range)	100 (89–100)	100 (0–100)	100 (0–100)
Family history of seizures	n = 212; p = 0.688	N (%)	11 (31.4)	50 (28.2)	61 (28.8)
PPH of depression	n = 211; p = 0.224	N (%)	0 (0.0)	12 (6.8)	12 (5.7)
PPH of psychotic episodes	n = 212; p = 1.000	N (%)	1 (2.9)	5 (2.8)	6 (2.8)
PPH of mental retardation	n = 212; p = 1.000	N (%)	4 (11.4)	23 (13.0)	27 (12.7)

1) Gwa...Generalised seizures that started within a specific age range (seizures most likely due to idiopathic epilepsy), Gfs...Generalised seizures with obvious focal neurological signs, Goa...Generalised seizures that started outside the age range of idiopathic epilepsies but without any obvious sign or history of an underlying cause, Gbd...Generalised seizures with more widespread brain damage, Tt...Two different seizure types, U...Unclassified epileptic seizures; for more details see Winkler et al. [19] PWE... People with epilepsy, NCC... Neurocysticercosis, Tx... Anti-epileptic treatment, PPH... Past psychiatric history.

People with active NCC lesions (n = 6) with an average age of 47.7 years (SD 23.4) were significantly older than people with inactive NCC (n = 25) with an average age of 25.2 years ((SD 15.5); Mann-Whitney-U, p = 0.015). Also, the mean age of PWE with active NCC at first seizure (41.2 years (SD 23.0)) was significantly higher when compared to PWE with inactive NCC (16.8 years (SD 16.5); Mann-Whitney-U, p = 0.007).

### Clinical Characteristics

The seizure frequencies, compliance with anti-epileptic drug (AED) and reduction of seizure frequency after treatment in PWE with and without NCC are listed in Table 1. There was a trend towards generalised epilepsies without focal neurological signs that started outside the age range of idiopathic epilepsies (implying that there may be structural brain damage) and generalised epilepsies with clear focal signs being commoner in the NCC group. On the other hand, idiopathic epilepsies, which are usually generalised seizures starting within a specific age range, and epilepsies associated with brain damage were more frequent in PWE without NCC.

The compliance with AED was slightly better in the group without NCC, although this did not reach significance. The administration of the type of AED was similar in both groups. Among compliant PWE taking their AED regularly, the group of PWE with NCC had a significantly higher reduction of seizures (Table 1). The percentage of people without seizures since their last visit was higher among compliant PWE (compliant: 54.1%, 59/109; non-compliant: 40.6%, 41/101; Fisher's exact test p = 0.054). Dividing compliant and non-compliant PWE according to the presence of NCC a higher percentage of people without seizures was observed among compliant PWE with NCC compared to those without NCC (compliant: 78.6%, 11/14 PWE with NCC, 50.5%, 48/95 PWE without NCC, Fisher's exact test p = 0.082; non-compliant: 42.1%, 8/19 PWE with NCC, 40.2%, 33/82 PWE without NCC, p = 1.000), although the result did not reach significance. In terms of past psychiatric history, family history of seizures and educational level no significant differences were found. For more details see Table 1.

### Risk Factors Associated with Neurocysticercosis

Number of people in household as an indicator of crowding, number of pigs at home, close contact with pigs and use of latrines as possible risk factors for NCC were not significantly associated with NCC. However, the percentage of people who consumed pork was significantly higher in the group of people with NCC (Table 2).

**Table 2:** Frequency of potential risk factors of neurocysticercosis in people with epilepsy with and without neurocysticercosis.

Potential risk factors of NCC			PWE with NCC	PWE without NCC	Total
Number of people in household		N	35	174	209
p = 0.856		Mean (SD)	6.9 (3.0)	7.4 (4.2)	7.3 (4.0)
		Median (range)	6 (2–13)	7 (1–30)	7 (1–30)
Number of pigs in household		N	35	174	209
p = 0.370		Mean (SD)	1.3 (2.0)	1.1 (2.0)	1.1 (2.0)
		Median (range)	0 (0–10)	0 (0–10)	0 (0–10)
Close contact with pigs	n = 212; p = 0.351	N (%)	17 (48.6)	70 (39.5)	87 (41.0)
Pork consumption	n = 209; **p = 0.001**	N (%)	34 (97.1)	128 (73.6)	162 (77.5)
Use of latrine	n = 209; p = 1.000	N (%)	34 (97.1)	169 (97.1)	203 (97.1)

PWE... People with epilepsy, NCC... Neurocysticercosis.

### Serological Analysis of Serum Samples

All samples which were positive using the CWB were also positive using the CDC EITB. Additionally, seven samples that were negative in the CWB were positive in the CDC EITB, which was statistically significant (McNemar, p = 0.016). Five of these seven had multiple calcified lesions highly suggestive of NCC, one had multiple calcified lesions and one hypodense lesion and one had a normal CT. The percentage of a positive CDC EITB result in the group of PWE with NCC calcifications ( = highly suggestive of NCC) was much higher (52.2%, 12/23) than the percentage of the same group tested with the CWB (13.3%, 2/15; “sensitivity”, if CT was used as a gold standard). Even with the small number of samples that was analysed in both labs (n = 15), this approached significance (negative in both tests: 8, positive in both tests: 2, CWB negative, CDC EITB positive: 5; McNemar, p = 0.063). Concerning people with a normal CT, all samples (20/20) were negative with the CWB, whereas 91.3% (42/46) were negative with the CDC EITB (“specificity”, if CT was used as gold standard). The results of CSF samples were identical in CWB and CDC EITB.

### Diagnosis of Neurocysticercosis

Using the results of the CDC EITB as the serological gold standard, as suggested by Del Brutto et al. [21], [22], 17 people were classified with definitive NCC, versus 7, if the data obtained from the CWB had been used (Table 3). Similarly, the percentage of PWE with probable and definitive NCC was 16.5% (35/212) using the CDC EITB results, compared to 13.7% (29/212) if the CWB was used.

**Table 3:** Diagnosis of neurocysticercosis according to Del Brutto et al. using two different antibody tests.

Diagnosis of NCC*	Commercial Western blot				CDC EITB			
	n	Positive	Negative	nt	n	Positive	Negative	nt
No NCC	183 (86.3%)	0	20	163	177 (83.5%)	0	47	130
Probable NCC	22 (10.4%)	0	13	9	18 (8.5%)	6	11	1
Definitive NCC	7 (3.3%)	6	1	0	17 (8.0%)	17	0	0

*According to NCC diagnostic criteria of Del Brutto et al. [21], [22]

NCC... Neurocysticercosis, CDC EITB... Electroimmunotransfer blot developed by the Centers for Disease Control and Prevention, Atlanta, GA, USA, nt... Not tested.

### Association of Antibody Test Results with Number and Type of Neurocysticercosis Lesions

The results of both antibody tests together with the number of total NCC lesions on CT, including cysts and calcifications, are listed in Table 4. The number of NCC lesions was significantly associated with a positive serum antibody result (Mann-Whitney-U, CWB: p = 0.006, CDC EITB: p<0.001). People with more CT lesions had a higher chance of a positive antibody result. Probably due to the low number of samples, there was no significant association in CSF (Mann-Whitney-U, p = 0.537).

**Table 4:** Number and activity of neurocysticercosis lesions on cerebral computed tomography scan and cysticercosis antibody results.

Number of NCC	EITB CDC		Commercial Western Blot		CSF both tests	
lesions on CT scan*	Negative	Positive	Negative	Positive	Negative	Positive
0	42 (91.3%)	4 (8.7%)	20 (100%)	0 (0.0%)	0	0
1	7 (77.8%)	2 (22.2%)	0	0	0	0
2–4	5 (45.5%)	6 (54.5%)	6 (100%)	0 (0.0%)	3 (100%)	0 (0.0%)
≥5	4 (26.7%)	11 (73.3%)	8 (57.1%)	6 (42.9%)	3 (37.5%)	5 (62.5%)
Total	58 (71.6%)	23 (28.4%)	34 (85.0%)	6 (15.0%)	6 (54.5%)	5 (45.5%)
**Activity of NCC lesions on CT scan**						
Inactive	16 (55.2%)	13 (44.8%)	13 (92.9%)	1 (7.1%)	6 (85.7%)	1 (14.3%)
Active	0 (0%)	6 (100%)	1 (16.7%)	5 (83.3%)	0 (0%)	4 (100%)

*Including active and inactive lesions; NCC... Neurocysticercosis, CDC EITB... Electroimmunotransfer blot developed by the Centers for Disease Control and Prevention, in Atlanta, GA, USA, CSF... Cerebrospinal fluid, CT...Computed tomography.

Active NCC lesions were significantly associated with a positive antibody result in both tests for serum and CSF (Fisher's exact test, Serum CWB: p = 0.002; Serum CDC EITB: p = 0.022; CSF both tests: p = 0.015, see Table 4). The prevalence of cysticercosis antibodies detected by the CDC EITB among people with single lesions was 22.2% (2/9, “sensitivity”, if CT was used as gold standard). CWB was not performed in people with single lesions (Table 4). People with at least two lesions, when analysed with CDC EITB and CWB had antibodies in 65.4% (17/26) and 30% (6/20), respectively (“sensitivity”, if CT was used as a gold standard). Looking at individuals with a positive CSF result, all corresponding serum samples were positive in the CDC EITB and four out of five were positive in the CWB.

## Discussion

### General Aspects

To our knowledge this is the first study that systematically compares demographic data and clinical characteristics of PWE with and without NCC in sub-Saharan Africa. A differentiation between these two groups is important not only because of the possible treatment of active NCC, but also because of prevention and disease control programs for NCC.

There are several limitations of our study. The diagnosis of epilepsy was made up to three years prior to the performance of the CT scan and the collection of specimens. Hence the number of people with active NCC might be underestimated. Due to lack of EEG the diagnosis of epilepsy is based on clinical examination and thorough interviews of patients and relatives. Epileptic seizures that appear generalized may have a short focal start that clinically goes unnoticed. Simple partial seizures may not be diagnosed, because patients do not report to the hospital. As to serology, it would have been preferable, if cysticercosis serology had been performed in all PWE. This unfortunately was not possible in our study.

### Clinical Characteristics of People with Epilepsy and Neurocysticercosis

Demographic data about PWE in low income countries is limited. Singhvi et al. described the patterns of patients (n = 100) with intractable epilepsy at a tertiary center in India. All patients received at least two AEDs. In their study, the mean age was 23.2 years with a mean duration of seizures of 11.4 years and their mean seizure frequency was 12.4 seizures per month [29]. In our study, the mean age was higher with 28.2 years and the average age at first seizure was 17.7 years. The older age of our study population could be explained by the exclusion of children younger than ten years at time of diagnosis. The mean seizure frequency in our drug naïve PWE with 7.7 seizures per month was lower compared to the study of Singhvi et al.

The mean age of PWE with NCC in our study (32.5 years) is comparable to the mean age (28.6 years) of people with NCC in a large retrospective study of people with NCC treated in Houston, Texas, that included 202 individuals mainly presenting with seizures [30]. In our study, there was a trend that PWE with NCC were older and that they had their first seizure later compared to PWE without NCC. This may suggest that NCC should be considered as an underlying cause of epilepsy especially among PWE with a late onset of seizures. Other studies reported that NCC is the cause of late onset epilepsy in up to 50% [31]–[34]. However, a recent review revealed similar percentages of NCC associated epilepsy in the age group below 20 years and that of 20–54 years of age [10]. These data suggest that NCC has also to be considered as a cause of epilepsy in young PWE.

In our study, PWE with active NCC were significantly older than PWE with inactive NCC, which might be explained by a different immune response to the infestation with age. Porcine cysticercosis was first detected in our study area in the late 1980s and previously pig husbandry was not common [12]. If infestations caused more calcifications in younger people, the lower number of calcifications among older people could also be explained by the absence of NCC before the introduction of pig husbandry.

The gender in our study population of PWE was equally distributed, which is in accordance with another study in Tanzania [35] and with the conclusion of reviews about the prevalence of epilepsy in Asia [36] and sub-Saharan Africa [1], which did not find a significant difference regarding gender. In our study slightly more males had NCC, a result similar to the one reported by Nicoletti et al., who diagnosed 21 males and 14 females with NCC among 124 PWE [34].

It is remarkable that, although PWE with NCC were less compliant, they had a lower seizure frequency. The reduction of seizure frequency in compliant PWE was significantly higher in PWE with NCC compared to those without NCC. 78.6% of compliant PWE with NCC had no seizures, whereas this was only the case in 50.5% of compliant PWE without NCC. These observations not only suggest that the frequency of seizures due to NCC seems to decline faster over time, but also that anti-epileptic treatment with carbamazepine or phenobarbitone, which is available in most low income countries, is very efficient in controlling seizures due NCC. Del Brutto et al., who investigated 203 PWE with NCC, reported a similar seizure control rate of 83% [37].

The reason for the higher percentage of NCC among people of the Iraqw tribe might be that according to our observation pig farming is more common in that tribe compared to other tribes. This difference might disappear in future, because tribes are intermingling and pig husbandry seems to become abundant in all tribes.

The past psychiatric history and family history of seizures seem to be similar in PWE with and without NCC. In our study, the only significant risk factor associated with NCC was pork consumption. In this context, we want to stress that cysticercosis is a faecal-oral infestation and pork consumption is not a prerequisite for cysticercosis. The consumption of undercooked pork or the handling of infected pig meat could lead to a tapeworm carrier in the household. Lescano et al. showed that there is a significant cysticercosis seroprevalence gradient around a tapeworm carrier and that the risk for cysticercosis is high, if there is a tapeworm carrier in the household [38]. Other factors related to social status such as number of people per household or educational level and also pig contact or number of pigs per household were not significantly associated with NCC. The use of latrines, which has been identified as a risk factor for porcine cysticercosis [39], was almost omnipresent in our study population. The number of people who did not use a latrine (6/209) was too low to identify significant differences.

### Antibody Tests for Cysticercosis in People with Epilepsy and Neurocysticercosis

It is difficult to define sensitivity and specificity of serological tests for cysticercosis, because the gold standard for the diagnosis of NCC is not well defined. Probably the best available single examination to diagnose NCC in low income countries is a CT scan. Taking the CT result as a gold standard, the comparison between the two antibody tests for cysticercosis revealed a difference mainly in terms of sensitivity. Especially among people with lesions highly suggestive of NCC, which are mainly multiple calcified lesions, the CDC EITB had a higher sensitivity than the CWB. Antibodies were detected in 52.2% and 13.3% with the CDC EITB and the CWB, respectively. However, sensitivity was low compared to other studies that reported a sensitivity of 60% to 89% in sera with only calcified cysts [40], [41]. Rajshekhar et al., who studied NCC and epilepsy in India, showed a positive EITB result in 26.1% (12/46) of PWE with NCC lesions on CT scan [7]. This relatively low number could be explained by the high percentage of people with only one or two calcifications (81.3%). In Peru, Montano et al. detected antibodies in 46.7% (7/15) of PWE with NCC lesions on CT scan. The type of lesions however was not specified [6]. In active lesions, where the CDC EITB detected antibodies in all six samples and the CWB in five of six, the number of samples was too low to show significant differences. Our results confirm the high sensitivity of the CDC EITB in people with more than one viable NCC cysts [28], [40]. As expected the total number of active and inactive NCC lesions was positively correlated with a positive antibody result in both tests. It seems that a higher burden of disease leads more often to a detectable antibody response. When looking at PWE with normal CT, it seems that the specificity of the CDC EITB (91.3%) is lower compared to the CWB (100%). However, it has to be considered that cysticercosis may be present in other organs such as eye and subcutaneous/muscular tissue or that calcifications smaller than 10 mm may be missed because of the thickness of CT slices. The specificity of the EITB is very high, near 100%, with only a few anecdotal reports [42], [43] of false positive results since the time the test was introduced in 1989. Therefore, it seems probable that the four PWE with a positive CDC EITB and normal CT scan may have had some exposure to *T. solium* larvae in the past or may harbour cysts in other organs than the brain.

The analysis of CSF with CDC EITB did not give additional information regarding the diagnosis of NCC, because all CSF positive cases had also positive serum samples. Our results are in accordance with the study of Proaño-Narvaez et al, in which analysis of serum and CSF samples with a EITB for cysticercosis were equally sensitive and specific [44]. We conclude that an analysis of CSF may only be indicated, if a quantitative test is used in order to calculate a specific *T. solium* antibody index of a serum/CSF pair.

In summary, our study compares demographic and clinical characteristics of PWE with and without NCC in rural northern Tanzania, showing that PWE with NCC tend to be older with a later onset of seizures compared to those without NCC. Seizure frequency in compliant PWE with NCC, using AEDs available on site, seems to respond better than in compliant PWE without NCC. The only risk factor for NCC that could be identified is consumption of pork. In addition, the sensitivity and specificity of a commercially available western blot and the CDC EITB was tested in PWE with NCC showing a higher sensitivity of the latter, especially in PWE with calcifications.

## Supporting Information

Checklist S1 **STROBE checklist.**
(PDF)
