# Supplementary material for: A Cross-Sectional Study of People with Epilepsy and Neurocysticercosis in Tanzania: Clinical Characteristics and Diagnostic Approaches
Source: PLoS Negl Trop Dis. 2011 Jun 7;5(6):e1185. doi: 10.1371/journal.pntd.0001185 (PMC3110162; doi:10.1371/journal.pntd.0001185)
Supplement: Checklist S1 — STROBE checklist. (PDF) [file pntd.0001185.s001.pdf]

STROBE Statement—Checklist of items that should be included in reports of ***cross-sectional studies***

	Item No	Recommendation
**Title and abstract**	1	(*a*) Indicate the study's design with a commonly used term in the title or the abstract<br>**ANSWER: Title: "A Cross-sectional Study of People with Epilepsy and Neurocysticercosis in Tanzania: Clinical Characteristics and Diagnostic Approaches"**
		(*b*) Provide in the abstract an informative and balanced summary of what was done and what was found<br>**ANSWER: Done in the abstract.**
**Introduction**		
Background/rationale	2	Explain the scientific background and rationale for the investigation being reported<br>**ANSWER: Line 80-111**
Objectives	3	State specific objectives, including any prespecified hypotheses<br>**ANSWER: Line 40-41, 108-111**
**Methods**		
Study design	4	Present key elements of study design early in the paper<br>**ANSWER: The term cross-sectional study is already mentioned in the title and the study design is described in the abstract.**
Setting	5	Describe the setting, locations, and relevant dates, including periods of recruitment, exposure, follow-up, and data collection<br>**ANSWER: Study site Line 124-131, Recruitment period Line 134, Details on data collection: 146-151, 153-184**
Participants	6	(*a*) Give the eligibility criteria, and the sources and methods of selection of participants<br>**ANSWER: Figure 1 and 133-144**
Variables	7	Clearly define all outcomes, exposures, predictors, potential confounders, and effect modifiers. Give diagnostic criteria, if applicable<br>**ANSWER: Some outcomes such as seizure frequency are self explaining. Diagnostic criteria for NCC are explained in line 179-185, Reduction of seizure frequency is explained in line 196-198, diagnostic criteria are explained in line 136-138, NCC lesions and sereologic results are explained in line 153-177, compliance: 149-151**
Data sources/ measurement	8*	For each variable of interest, give sources of data and details of methods of assessment (measurement). Describe comparability of assessment methods if there is more than one group<br>**ANSWER: diagnosis of epilepsy: line 139-141, Standardised interview: 146-151, CT-variables: 154-166, serologic data: 167-177, There were no differences in assessment of the two groups.**
Bias	9	Describe any efforts to address potential sources of bias<br>**ANSWER: Potential bias are adressed in the first chapter of the discussion (line 283-290)**
Study size	10	Explain how the study size was arrived at<br>**ANSWER: See figure 1. Due to the lack of data, it was not possible to calculate the power in advance.**
Quantitative variables	11	Explain how quantitative variables were handled in the analyses. If applicable, describe which groupings were chosen and why

		**ANSWER: Line 189-191. The only grouping of quantitative variables was educational level based on the duration of primary and secondary school, which is described in table 1 (Line 551ff) and number of NCC-lesions in table 4, which was done according to the comments of a reviewer #4 in order to improve the information (see Table 4)**
Statistical methods	12	(*a*) Describe all statistical methods, including those used to control for confounding
		(*b*) Describe any methods used to examine subgroups and interactions
		(*c*) Explain how missing data were addressed
		(*d*) If applicable, describe analytical methods taking account of sampling strategy
		(*e*) Describe any sensitivity analyses<br>**ANSWER: a-d see line 187-195, e: sensitivity for serological tests: Lines 270-273, discussion since sensitivity is not clearly defined: line 350-354.**
**Results**		
Participants	13*	(a) Report numbers of individuals at each stage of study—eg numbers potentially eligible, examined for eligibility, confirmed eligible, included in the study, completing follow-up, and analysed
		(b) Give reasons for non-participation at each stage
		(c) Consider use of a flow diagram<br>**ANSWER for 13a-c: figure 1 and Line 133-144**
Descriptive data	14*	(a) Give characteristics of study participants (eg demographic, clinical, social) and information on exposures and potential confounders<br>**ANSWER: Line 204-213 and Table 1**
		(b) Indicate number of participants with missing data for each variable of interest<br>**ANSWER: We also used natural numbers to report categorical variables. Hence the number of participants with missing data can be calculated for every variable. In numeric variables the n is mentioned.**
Outcome data	15*	Report numbers of outcome events or summary measures<br>**ANSWER: Our study consists mainly of descriptive data. Outcome data such as seizure frequency or reduction of seizure frequency are reported in table 1 and line 222-231.**
Main results	16	(*a*) Give unadjusted estimates and, if applicable, confounder-adjusted estimates and their precision (eg, 95% confidence interval). Make clear which confounders were adjusted for and why they were included<br>**ANSWER: We only used unadjusted estimates in this study.**
		(*b*) Report category boundaries when continuous variables were categorized<br>**ANSWER: Category boundaries for educational level and number of NCC lesion are mentioned in table 1 and table 4**
		(c) If relevant, consider translating estimates of relative risk into absolute risk for a meaningful time period<br>**ANSWER: not relevant for our study**
Other analyses	17	Report other analyses done—eg analyses of subgroups and interactions, and sensitivity analyses<br>**ANSWER: Some subgroup analyses are reported throughout the results and all tables and are explained. Sensitivity analyses are mentioned and discussed in Lines 270-273 and 350-354.**
**Discussion**		
Key results	18	Summarise key results with reference to study objectives

		**ANSWER: line 279-282 and 382-388**
Limitations	19	Discuss limitations of the study, taking into account sources of potential bias or imprecision. Discuss both direction and magnitude of any potential bias<br>**ANSWER: lines 283-290**
Interpretation	20	Give a cautious overall interpretation of results considering objectives, limitations, multiplicity of analyses, results from similar studies, and other relevant evidence<br>**ANSWER: Done throughout Discussion (line 278-388)**
Generalisability	21	Discuss the generalisability (external validity) of the study results<br>**ANSWER: Done throughout Discussion (line 278-388)**
**Other information**		
Funding	22	Give the source of funding and the role of the funders for the present study and, if applicable, for the original study on which the present article is based<br>**ANSWER: Sources of funding were listed during the submission process and as far as we know will be mentioned on the side. To avoid reiteration we did not mention that in the text. The funders had no role in study design, data collection, analysis and publication. There are no conflicts of interest.**

*Give information separately for exposed and unexposed groups.

Note: An Explanation and Elaboration article discusses each checklist item and gives methodological background and published examples of transparent reporting. The STROBE checklist is best used in conjunction with this article (freely available on the Web sites of PLoS Medicine at http://www.plosmedicine.org/, Annals of Internal Medicine at http://www.annals.org/, and Epidemiology at http://www.epidem.com/). Information on the STROBE Initiative is available at www.strobe-statement.org.
